# Optimising Psychosocial Interventions for People With Severe and Enduring Eating Disorders (SEED) Perspectives of Healthcare Professionals in Ireland: A Qualitative Study

**DOI:** 10.1111/inm.70037

**Published:** 2025-04-07

**Authors:** Kate Mooney, Ann‐Marie Bright, Annmarie Grealish

**Affiliations:** ^1^ School of Nursing, Health Research Institute University of Limerick Limerick Ireland; ^2^ Florence Nightingale Faculty of Nursing, Midwifery & Palliative Care King's College London London UK

**Keywords:** healthcare professionals, psychosocial interventions, qualitative research, severe and enduring eating disorder, thematic analysis

## Abstract

To date, no studies have explored healthcare professionals' perceptions on the acceptability and effectiveness of psychosocial interventions for patients with severe and enduring eating disorders in Ireland. The aims of this study were to explore how healthcare professionals view the use of psychosocial interventions for patients with severe and enduring eating disorders and how these approaches can be improved. A qualitative study design was utilised to explore perceptions and opinions. Semi‐structured, one‐to‐one interviews were used to collect data from healthcare professionals (*n* = 16) in mental health settings. Interviews were audio‐recorded and transcribed verbatim before being analysed using thematic analysis. Four themes were identified: (1) therapeutic relationship, (2) patient‐centred approaches, (3) co‐morbidities and (4) pathway of care and services. Findings strongly suggest the need for patients with severe and enduring eating disorders to set their own goals to improve treatment outcomes and quality of life. Furthermore, findings also suggest a strong correlation between a history of trauma and the diagnosis of severe and enduring eating disorders. Therefore, training for healthcare professionals to provide trauma‐informed care is needed.

## 1 Introduction

Eating disorders (EDs) are characterised by a severe and persistent disturbance in eating and related behaviours (including severe dietary restriction, binge eating, self‐induced vomiting, laxative misuse and excessive exercise) that cause psychological and physical impairments (Keski‐Rahkonen and Mustelin 2016). EDs have the highest rates of mortality of all mental health disorders, and although remaining difficult to treat, recovery is possible with effective treatment (Fichter and Quadflieg 2016; Yao et al. 2021; Yager 2020). The National Clinical Programme for Eating Disorders in Ireland (Health Service Executive [HSE] 2018) estimates there are approximately 1800 new cases of EDs every year. Indeed, the Medical Emergencies in Eating Disorders (MEED) guidelines identify that between 2018 and 2022, hospital admissions for EDs increased by 84% (Royal College of Psychiatrists 2022). Data from the Health Research Board identified hospital admissions for adults with EDs in Ireland increased from 138 in 2019 to 210 in 2022 (Daly and Craig 2022).

Despite the availability of effective treatments for people with EDs, the recovery rate remains low, with less than half of those affected achieving recovery (Yao et al. 2021; Edakubo and Fushimi 2020; Reay et al. 2022). The term Severe and Enduring Eating Disorder (SEED) is often used for patients with longstanding EDs and has been distinguished using criteria such as an illness duration of ≥ 3–7+ years, clinical severity and being the recipient of evidence‐based treatments with little or no benefit (Hay and Touyz 2018). However, an individual may have a long‐standing ED that is not severe and vice versa, highlighting the lack of clarity that exists when categorising individuals as experiencing SEED (Broomfield et al. 2021; Reay et al. 2022).

## 2 Background

Over 70% of individuals with EDs are reported to have co‐morbidity such as trauma symptomatology, self‐harm, anxiety, mood disorders and substance use (Keski‐Rahkonen and Mustelin 2016; Yager 2020). Several studies have made the connection between individuals with EDs and traumatic histories and post‐traumatic stress disorder (PTSD) (Scharff et al. 2021a; Scharff et al. 2021b; Rabito‐Alcón et al. 2021; Brewerton et al. 2020, 2022; Brewerton 2023). These studies showed that individuals with trauma histories experience a higher severity of ED symptoms, higher suicidality, higher anxiety, more depressive symptoms and an overall poorer quality of life. Palmisano et al. (2016) list these traumatic experiences to include emotional, physical or sexual abuse, emotional or physical neglect, being bullied by peers, being witness to domestic abuse and serious accidents. Other studies highlighted the progress made in the intersection between trauma/PTSD and EDs and showed how patients achieved sustained improvements in ED symptomology after completing an integrated, multimodal clinical approach while also gaining long‐term significant reductions in PTSD symptoms (Mitchell et al. 2021; Trottier and Monson 2021; Claudat et al. 2022; Perlman 2023).

A growing body of literature highlights the need for healthcare professionals (HCPs) to better recognise the common co‐morbidity between EDs, trauma, PTSD and autism spectrum disorder (ASD) to improve response to treatments, help support complex conditions and improve quality of life (Brewerton et al. 2020; Mitchell et al. 2021; Tchanturia 2022; Reay et al. 2022). Approximately 20%–30% of individuals with EDs meet the diagnostic criteria for ASD. Indeed, Kinnaird et al. (2017) and Westwood and Tchanturia (2017) attributed this co‐morbidity to poorer response and outcomes to treatments. These studies demonstrated how ASD traits were more prevalent in individuals with EDs and may exacerbate psycho‐pathologic characteristics such as rigid thinking, behavioural, cognitive and social difficulties. Similarly, Babb et al. (2021) found that autistic traits were often dismissed in ED services or misjudged as behaviours driven by having an ED. An example of this was when fidgeting was perceived as an attempt to burn calories. Communication difficulties were also misidentified as the patient disengaging from their treatment, and HCPs reported they found it difficult to disentangle whether behaviours were linked to autism or an ED.

The National Institute for Health and Care Excellence (NICE) (2017) recommends early‐stage psychotherapy at outpatient level for individuals with EDs; however, there is a lack of guidelines for the treatment of SEEDs. Countries such as Australia and New Zealand provide clear and comprehensive guidelines for this cohort, including maintaining and instilling hope, choosing a harm minimisation approach and improving quality of life (Hay et al. 2014; Westmoreland and Mehler 2016). The Royal College of Psychiatrists (2022) highlights the need for HCPs to be trained to recognise the early stage of EDs to help prevent potential delays in treatment and the development of a long‐standing ED. Intensive day and home‐based treatments are increasingly recognised as effective and more cost‐effective alternatives to inpatient care, which usually involves individual and family psychosocial interventions (PSIs) and nutritional therapy (PwC 2015; Striegel Weissman and Rosselli 2017; HSE 2018; Hay et al. 2019; National Health Service [NHS] 2019). Evidence‐based PSIs such as Cognitive Behavioural Therapy‐Enhanced (CBT‐E), Maudsley Anorexia Nervosa Treatment for Adults (MANTRA), Specialist Supportive Clinical Management (SSCM) and Compassion‐Focused Therapy for Eating Disorders (CFT‐E) are recommended and effective for treating adults with EDs (Atwood and Friedman 2020; Costa and Melnik 2016; de Jong et al. 2020; Raykos et al. 2018; Wildes et al. 2017). The core principles of each psychosocial intervention are outlined in Table 1. These PSIs help to address the social and psychological factors involved in the onset and maintenance of the ED, and HCPs therefore require access to a range of psychosocial treatment approaches to meet the varied needs of this cohort of patients. Ross (2017) suggests that to be able to provide effective treatment for patients with an ED, it is crucial to explore the root cause of the ED, as this can often be related to unresolved or untreated trauma.

**TABLE 1:** Overview of the psychosocial interventions used for the treatment of SEED.

Psychosocial intervention	Core principles
Cognitive Behavioural Therapy‐Enhanced (CBT‐E)	Transdiagnostic treatment for all forms of ED Highly individualised Four stages: Stage 1: starting well, Stage 2: taking stock, Stage 3: body image (dietary restraint, events moods and eating, mindsets) and Stage 4: ending well Typically provided over 20 sessions, lasting 50 min each
Maudsley Anorexia Nervosa Treatment for Adults (MANTRA)	Cognitive interpersonal treatment Manualised approach to the factors that maintain EDs Focuses on thinking styles, emotional and relational styles, value of AN and responses from others Two‐pronged formulation using letter and diagram
Specialist Supportive Clinical Management (SSCM)	Combines clinical management (focussed on weight restoration and normal eating) and psychoeducation Supportive psychotherapy Two broad aims: to help identify links between symptoms and eating behaviour/weight and to support a gradual return to normal eating and weight restoration
Compassion‐Focused Therapy for Eating Disorders (CFT‐E) CFT‐E	Teaches skills to recognise and regulate emotions Enhances compassionate motivation for change Addresses thoughts and feelings linked with eating Helps to bring about clinically significant change in behaviours

Treem et al. (2023) recommend a palliative care model for patients with SEED who have not responded to evidence‐based models. Palliative care aims to support the individual's personal goals for their care, reduce physical distress and improve social functioning. However, more integrated concurrent treatments are needed to provide people with strategies and coping skills to self‐manage symptoms, maintain recovery and stay well for longer periods (Calugi et al. 2017; Eddy et al. 2017; Russell et al. 2019; Yager 2021; Reay et al. 2022).

Despite the wide range of treatments recommended by NICE (2017), less than half of individuals with EDs recover. Recent studies (Yager 2021; Reay et al. 2022) highlighted how 20%–30% of people with EDs do not recover and instead progress to having more complex and extensive treatment histories, multiple admissions to hospital, poorer quality of life and high mortality rates. These studies highlight the need to understand how HCPs are optimising and providing evidence‐based PSIs to patients with SEED. To the best of our knowledge, this is the first Irish study to explore HCPs' experiences of current approaches to PSIs for people with SEED and how current PSIs can be improved. Our study aimed to answer the following research question: ‘What are the perspectives and opinions of Irish healthcare professionals in providing psychosocial interventions to people with SEED?’

The objectives of this study were:

1. To interview healthcare professionals on their experiences of using current PSIs with patients with SEED.
2. To identify the characteristics, barriers and enablers of PSIs currently used with patients with SEED and how these could be improved.
3. To identify how HCPs are assessing and treating co‐morbidity such as traumatic histories and/or comorbid PTSD trauma in patients with SEED.
4. To identify the support, competency skills and training requirements for delivering PSIs for patients with SEED, including integration of trauma‐informed care.

## 3 Methods

### 3.1 Study Design

A descriptive qualitative study design and reflexive thematic analysis, guided by Braun and Clarke's framework (Braun and Clarke 2006) was used to explore HCPs perspectives and experiences of working with patients who have SEED within mental healthcare settings, and the use of PSIs from multiple perspectives. The Consolidated Criteria for Reporting Qualitative Research (COREQ) 32‐item checklist (File S1) was followed to enhance the transparency of our study's reporting (Tong et al. 2007).

### 3.2 Ethics

Ethical approval was obtained from the three Health Research Ethics Committee sites in Ireland (Reference numbers: CH09—REC no: 11/2023; REC no: 2024‐007; REC no: 989).

### 3.3 Study Setting and Selection of Participants

One‐to‐one, semi‐structured interviews were conducted with HCPs from nine mental health/ED services in Ireland. All participants were purposively sampled by key characteristics to capture diversity in mental health settings/ED services. The eligibility criteria used to purposefully select each participant are illustrated in Table 2, and snowball sampling (Sadler et al. 2010) was additionally used to increase the number of participants.

**TABLE 2:** Eligibility criteria for participants.

Types of participants	Inclusion criteria	Exclusion criteria
Healthcare professionals	– Being a registered HCP with a professional body (e.g., Nursing and Midwifery Board of Ireland [NMBI]) – Have a minimum of 1‐year clinical experience working with patients with SEED – HCPs who are currently treating patients with SEED – Able to provide informed consent	– HCPs who do not work with patients with SEED – Unable to give informed consent

### 3.4 Recruitment and Sampling

The recruitment process took place over a 3‐month period from January 2024 to March 2024. The principal investigator (PI) (KM) contacted potential HCPs and introduced the study in nine sites. All potential participants were provided with the Participant Information Leaflet (PIL) detailing the study's purpose, their rights in relation to confidentiality and the voluntary nature of participation, and gave time for HCPs to express interest, of which 16 (*n* = 16) HCPs agreed to participate. The PI screened all participants who expressed interest in the study against the eligibility criteria and obtained formal consent via email and in person prior to all interviews being conducted.

### 3.5 Patient and Public Involvement (PPI)

A PPI group was set up to enable experiential knowledge to collaborate in the research process, as well as to improve the study's quality and relevance to HCPs participants. Two HCPs experienced working with SEED were involved in this study as research advisors. The PPI advised and gave feedback on the interview questions, language used with HCPs participants and collaborated on the recruitment process.

### 3.6 Data Collection

Individual, semi‐structured interviews were conducted and audio‐recorded by the PI (KM) in person and on Microsoft Teams between January 2024 and April 2024. The semi‐structured interview topic guide (File S2) was informed by the current evidence in the literature (Reay et al. 2022) and further developed with the study's PPI representatives. The study's topic guide was initially piloted with the PPI group and clinical colleagues within the ED services, which resulted in minor adjustments to the flow and order of the questions. Additionally, participants' demographic information was obtained through online questionnaire via Qualtrics prior to interview. Participants were also asked to complete the Evidence‐Based Practice Attitude Scale (EBPAS) (Aarons 2004) prior to interviews as this helped to further validate and strengthen the qualitative data. The EBPAS‐15 is a self‐report validated instrument which demonstrated good reliability and internal consistency (Cronbach's alpha 0.76) (Aarons et al. 2010). This scale measures HCPs' attitudes towards adopting new treatments, interventions, therapies and practices in mental health settings (Aarons et al. 2010). The four subscales assesses mental health service providers' attitudes towards the adoption of evidence‐based practices: (1) openness to implementing new interventions (Openness); (2) the intuitive appeal of the new intervention (Appeal); (3) willingness to use required interventions (Requirements) and (4) conflict between clinical experience and research results (Divergence). Responses to scale items are measured on a 5‐point Likert scale ranging from 0 (*Not at all*) to 4 (*To a very great extent*). The four subscales on the EBPAS combine into a higher order total scale score representing respondents' global attitudes towards adopting the evidence‐based practice. For the purpose of this study, the mean score on the total scale score was computed in order to assess the participant's attitudes towards adopting new psychosocial interventions in supporting patients with SEED.

### 3.7 Data Analysis

All interviews were transcribed verbatim and anonymised by the PI (KM) and imported into NVivo (Version 14) and then stored in a password‐protected database for data management purposes. The PI (KM) also kept a reflective diary and notes during and after interviews, which aided the analytic process. Reflexive Thematic Analysis (Braun and Clarke 2021) was used to guide the researcher's lens through personal experiences and prior knowledge of working with patients who have SEED within mental healthcare settings. Braun and Clarke's (2006) six‐step process of thematic analysis was used to extrapolate and analyse the data from the interview transcriptions. The PI (KM) undertook the initial analysis by conducting all the initial coding. The final themes were then discussed and reviewed with the other two authors (AMB and AMG) to explore interpretation and their relevance to answering the research question and study objectives. The authors then chose relevant participant quotes to accentuate the themes. To enhance analytical rigour, the four criteria of qualitative research proposed by Lincoln and Guba (1985) (credibility, dependability, confirmability and transferability) was used (see Table 3).

**TABLE 3:** Trustworthiness of the qualitative data as recommended by Lincoln and Guba (1985).

Quality criteria	Action taken by the researchers
Credibility	Including HCPs who care for patients with SEED HCPs were recruited from inpatient and community settings Use of quotes to support the findings and ensure data accuracy
Dependability	Detailed description of the study methodology Application of reflexive thematic analysis discussed in the data analysis section The PI (KM) kept a reflexive diary and took field notes during and after interviews which included notes on theme identification and what was influencing them (Braun and Clarke 2021) Keeping a record of the coding tree and thematic map to guide the development of the themes and subthemes that emerged from the data
Confirmability	The transcripts were read independently by all authors—this ensured the appropriateness of the codes Thematic analysis was applied as described in data analysis Codes, themes and subthemes were discussed among the researchers to avoid bias in interpretation and inter‐coder agreement was used to confirm the final themes and subthemes
Transferability	The use of verbatim transcriptions were used to ensure the accuracy of the data Descriptions of the research setting in which the participants were recruited The characteristics of the study participants were provided
Reflexivity	As proposed by Braun and Clarke (2021), reflexivity was used that increased the quality in the qualitative evidence The interpretation of the data was questioned to ensure data accuracy and avoid interpretation bias A reflective diary was maintained by the PI (KM)

## 4 Findings

### 4.1 Participant's Characteristics

An overview of the participant characteristics is presented in Table 4. A total of 616 min of interview content was collected, median interview length was 40 min (range from 17 to 94 min). Fifteen participants were female, one participant was male, and they were aged between 34 and 65 years (mean ages was 44.8). Participants were from a diverse range of disciplines that included mental health nurse specialists (*n* = 8, 50%), clinical psychologists (*n* = 4, 25%), dieticians (*n* = 2, 12.5%), social workers (*n* = 1, 6.25%) and consultant psychiatrists (*n* = 1, 6.25%). The average number of years working in clinical practice ranged from 8 to 35 years and mean duration working with patients with SEED was 7.67 years. The EBPAS‐scale was completed by all participants (*n* = 16) prior to the interview, mean score 37.57 (SD 6.40) (Table 4) which strongly indicated that participants were adaptable to new evidence‐based practice/interventions in supporting patients with SEED.

**TABLE 4:** Demographic characteristics of participating healthcare professionals.

Characteristics of participants (total *n* = 16)	
Ages in years, mean (SD)	
Age range 30–65 years	44.8 (SD 9.81)
Gender, *n* (%)	
Female	*n* = 15 (93.75%)
Male	*n* = 1 (6.25%)
Ethnicity, *n* (%)	
White Irish	*n* = 14 (87.5%)
Asian	*n* = 1 (6.25%)
Eastern European	*n* = 1 (6.25%)
Professional role, *n* (%)	
Registered psychiatric nurse registered on Nursing and Midwifery Board of Ireland (NMBI)	*n* = 8 (50%)
Registered clinical psychologist	*n* = 4 (25%)
Registered dietician	*n* = 2 (12.5%)
Registered social worker	*n* = 1 (6.25%)
Registered consultant psychiatrist	*n* = 1 (6.25)
Educational attainment, *n* (%)	
Post graduate diploma (PGDip)	*n* = 7 (47.35%)
Masters (MSc) degree	*n* = 4 (25%)
Doctorate in Clinical Psychology (D.Clin.Psych)	*n* = 4 (25%)
Doctor of Philosophy (PhD)	*n* = 1 (6.25%)
Years in role as HCP, mean (SD)	
Range 8–35 years	18.4 (7.07)
Years' experience working with patients with SEED, mean (SD)	
Range 1–20 years	7.67 (4.92)
Evidence‐Based Practice Attitude Scale^a^, mean (SD)	37.57 (6.40)

^a^ The Evidence‐based Practice attitude Scale – Nurse (EBPAS), assessed the healthcare professional's attitudes towards adopting new psychosocial interventions in supporting patients with SEED.

**FIGURE 1:**
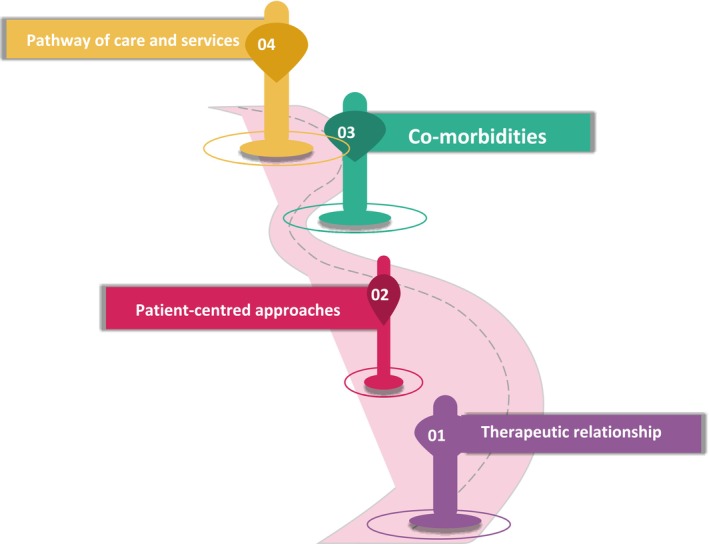
Four themes.

Four overarching themes (Figure 1) were identified from the participant data: (1) therapeutic relationship, (2) patient‐centred approaches, (3) co‐morbidities and (4) pathway of care and services.

#### 4.1.1 Therapeutic Relationship

All participants emphasised that the therapeutic relationship was vital when working with patients with SEED. Trust and empathy were considered integral factors to building a good rapport. The participants were of the view that it was essential to meet patients ‘where they are at’ and to collaboratively work on a treatment plan they have chosen:

I think building the relationship is key. If you can't build the therapeutic relationship… the therapies can go out the window. (Social Worker)

HCPs were all clear that facilitators to building the therapeutic relationship included giving patients with SEED the *‘space’* to discuss their story. Participants discussed the importance of getting to know the person and not just focusing on the ED diagnosis or the clinical presentation (e.g., low BMI, dietary intake and bone density) as patients with SEED will be ‘frustrated with hearing this time and time again’. HCPs noted that a better approach was to make it clear that they are not trying to rid the individual of their ED nor asking them to make changes they were not ready to make. Instead, the participants discussed the need to give patients the space to explore the function the ED serves and work on building alternative coping strategies. One participant stated:

[you] have to get to know the client first… what their likes is, a little bit of their own life, their dislikes… have a conversation about them, not about their ED… (Clinical Nurse Specialist)

Potential barriers to the therapeutic relationship may exist for patients who have had an ED diagnosis for a prolonged period and have previous negative experiences with services. HCPs acknowledged attending services can be traumatising in itself as they physically automatically react to negative memories which are often associated with feeling forced to do something they did not want to do (e.g., give up their ED diagnosis/identity, weight restore and unwanted inpatient admission). HCPs reported that these negative experiences were associated with feelings of failure, with one participant stating:

…fear, their threat systems being activated at the notion that something is going to be taken away… that they're going to kind of lose their autonomy… their choice within that relationship. (Psychologist)

#### 4.1.2 Patient‐Centred Approaches

Participants discussed the need for a patient‐centred approach to care for patients with SEED. Participants reported how traditional approaches to care can have a disempowering effect and often lead to patients disengaging from treatment, as it reinforces feelings of low self‐worth (e.g., feelings of failure, unable to improve/recover). Instead, participants highlighted the need to formulate their own goals with this population instead of focusing solely on weight restoration. One participant stated:

You're going from the non‐expert position… but if it's not coming from them or they're not ready… it's not going to work. (Social Worker)

Participants emphasised various therapeutic interventions that they facilitate for patients with SEED, including Cognitive Behavioural Therapy‐Enhanced (CBT‐E) which is specific to eating disorders, the Maudsley Model of Anorexia Nervosa Treatment for Adults (MANTRA), Specialist Supportive Clinical Management (SSCM), Schema Therapy for EDs and Compassion‐Focused Therapy for Eating Disorders (CFT‐E). Collectively, participants felt that CBT‐E was not the most effective choice when used for this cohort, as it requires patients to be ready to make behavioural changes from the outset.

It's highly demanding and I don't think it's fair to expect somebody who has a really long history of an ED and has a really strong attachment to it, to be in a position to be able to kind of jump into CBTe. (Psychologist)

MANTRA and Schema Therapy were described as giving patients more space to explore the function of the ED and what was maintaining their ED. Participants felt this allowed time to discuss with patients any ambivalence towards change. CFT‐E was noted to be an optimal choice, as patients with SEED were often self‐critical and experiencing low self‐worth coupled with experiencing embarrassment around their behaviours and feeling hopeless regarding treatment. Participants noted the importance of exploring the root cause of the patient's ED and empathetically acknowledging and normalising the reasons they may not feel able to make change (e.g., past trauma). Family therapy was also identified by the participants as beneficial, if the patient was open to this. HCPs emphasised the importance of specific training on perfectionism and thinking styles. However, as ED‐specific therapies emphasise weight restoration, participants were of the view that more focus should be put on supportive sessions and reducing expectations with clear achievable goals chosen by patients with SEED.

HCPs described the importance of focusing on improving quality of life for patients with SEED. Participants were also of the view that approaches such as regular physical monitoring should be prioritised to maintain physical risk to a stable level where patients have reduced discomfort and are stable enough to remain out of hospital and engage with community services. This highlighted the value of having a Multidisciplinary Team (MDT) and links with the General Practitioner (GP). HCPs reported that low BMI and physical complications, can become chronically stable and therefore PSIs should be offered unless there is immediate physical risk. HCPs reported that often patients with SEED do not want to give up their ED as it has become a part of their identity and described how their role involves supporting patients to find the optimal way of ‘living with’ SEED. HCPs favoured the social model as it focuses on integrating into society through social connection, for example, ‘having coffee in a cafe with a friend’. From a dietetic perspective, it was emphasised that often patients with SEED will not want to weight restore and expectations need to be adapted for this population. Instead, support should be directed at ‘making tweaks to nutritional intake’ that may reduce discomfort and improve quality of life. This will be a collaborative goal agreed with the patient and usually focuses on the areas of high risk, such as stabilising bloods, reducing laxative use to adding fibre to prevent gastroparesis.

For us to have… flexibility to be person focused… collaborative in how we define a good outcome… how we define change… develop goals with the service user around things that might have a positive impact on them… doesn't necessarily equate to recovery but is meaningful. (Psychologist)

HCPs highlighted the importance of being part of an MDT and highlighted that MDTs need to be adequately resourced with HCPs to provide diverse discipline support and to allow HCPs to support one another within the team through sharing knowledge, de‐briefing and team building. HCPs emphasised the importance of one‐to‐one/group supervision and treatment model specific supervision. There was a strong focus on the value of having an Occupational Therapist (OT) to focus on relational/social aspects and exploring interests outside of having an ED. The view of this approach would be that if a patient feels they have a better quality of life or hobbies to engage in, they may not need their ED as much.

… the occupational therapist and the social worker play in their psychosocial interventions… not necessarily evidence‐based from an ED perspective… offer something that can be really meaningful to the service user, quality of life focused, increasing day‐to‐day functioning, enhancing relationships, educate family…and partners about what's helpful and unhelpful… how to support individuals and empower them… those kinds of interventions… in the direction of recovery but creating… meaningful change. (Psychologist)

Although improving quality of life may be a form of recovery for some individuals, some participants felt it does not meet the criteria for an individual to be considered recovered from their ED.

……you can work on quality of life but until you work on the behaviours, you're not in the realm of recovery and to say that you are is in some way colluding with the ED. (Psychologist)

#### 4.1.3 Co‐Morbidities

HCPs felt that assessing for co‐morbidities was a priority to support psychological adaptation in living with SEED. However, they described how this was often ‘overlooked’ by HCPs because a greater focus is placed on stabilising patient's physical condition (BMI, weight etc.) and managing their ED psychopathology. HCPs identified trauma and autism as common co‐morbidities that patients with SEED present with and highlighted the detrimental long‐term effect these can have on them, for example, ‘attachment difficulties’, ‘difficulties developing and maintaining relationships’ and ‘poor family dynamics’. HCPs reported how patients often use their ED as a coping strategy to manage unresolved trauma in their life or flashbacks that may be associated with PTSD which can often leave them feeling ‘stuck’ and not managing to make behavioural changes. HCPs indicated if they helped treat the trauma this would support patients with their symptoms of SEED and emphasised how a holistic approach was needed to support patients to make the connection between their trauma(s) and SEED. Instead, these are often treated in isolation and co‐morbidities such as trauma and ASD are often overlooked. Instead, the focus is on treating the SEED and not what ‘has happened’ to the patient.

However, most HCPs did report that they were not equipped to work with trauma and felt they did not have the necessary time, training or skills to assess and effectively manage trauma with patients with SEED, stating ‘we wouldn't have adequate training to deal with PTSD, that's more of a psychologist domain’ and ‘we certainly could be upskilled now in kind of dealing with trauma because it's very difficult to treat other stuff when there's so much trauma going on’. One participant stated:

Anyone who works with ED needs to have skills to deal with trauma… even living with an ED for so long is very traumatising… you lose people… families burn out and that is… perceived as a rejection or trauma… (Psychologist)

HCPs reported the challenges they faced when identifying and/or differentiating symptoms of an ED presentation and autism. HCPs reported a definite preference for ‘expert’ OT skills to manage rigidity in routine, cognitive thinking styles and sensory issues that are often associated with autism; ‘there's an even greater need for that team approach’. HCPs were of the view that autism hinders recovery and indicated ASD could possibly attribute to development of SEED. HCPs emphasised that they need to be adequately trained and equipped to take an individualised approach and adapt ED treatment appropriately for these patients;

… when they got their autism diagnosis… I'd be wondering the validity of the diagnosis… for example, someone who is very low weight can present as having autism and they don't, or the ED is part of the autism. (Psychologist)

#### 4.1.4 Pathway of Care and Services

HCPs highlighted that there are not sufficient treatments or an appropriate pathway of care for patients with SEED. HCPs emphasised that inpatient/residential care was not suitable, particularly for those who do not want inpatient care, due to the high demands involved regarding weight restoration, dietary intake and lack of autonomy. These challenges can result in feelings of failure which re‐enforces beliefs that patients cannot be without their ED. Instead, HCPs felt treatment should be facilitated in the community by creating individualised care plans with goals that address how the patient wants to live their lives and function on a day‐to‐day basis. HCPs suggested having more emphasis on PSIs to help bring about behavioural and social changes where they can ‘meet people in similar situations’, ‘make connections and engage in peer‐support’. The overall consensus was that support should not be time‐limited and a place should be available where the patient can attend to have a check‐in/review, group support and work on their own goals with their keyworker. A Clinical Nurse Specialist described what this approach might look like;

You can drop‐in, like community groups for example, like a breastfeeding group… you don't have to go all the time, but it's based in your community. Your public health nurse is there, you get support from other people… in similar situations… not something that you have to do… you can go have a cup of coffee and talk about nothing… doesn't have to be about the ED… if there's an issue you can speak to someone. (Clinical Nurse Specialist)

However, for patients engaging in evidence‐based treatments, HCPs were of the view that blocks of psychological therapy, although individualised, should be time‐limited with an emphasis on having regular reviews of progress and engagement. Participants were also of the view that all ED clinicians should have training in all front‐line ED‐specific therapies. However, they stated that more research was needed to explore new and improved therapies for patients with SEED and to receive training in ‘lived experience’. Upcoming psychedelic therapy trials were mentioned as an area of interest:

They talked about psychedelic therapy… maybe we need to just be more flexible, like curious and open… to what else is out there… what we have at the moment is not really good enough for that population. (Psychologist)

Communication styles and use of appropriate language were repeatedly highlighted as an area that HCPs need to have increased awareness in. HCPs were of the view that more training for mental health professionals was required at university level and more specialist training was needed for all mental health nurses, GPs, general nurses, medical staff and HCPs working in Community Mental Health Teams (CMHTs) and primary care in PSIs for the treatment and management of SEED.

## 5 Discussion

The therapeutic relationship, particularly trust, was identified in this study as critical when supporting patients with SEED. Werz et al. (2022) found that patients who are chronically ill often show a high ambivalence to treatment and benefit from a trusting therapeutic relationship. This study found that although having a strong therapeutic relationship may not always lead to behavioural change, it is valuable for the patient to have social connection and feel understood. This study also found that patients benefited from having a sense of choice and control living with their SEED as ED is their identity and therefore are scared to be without it. Similarly, Gregertsen et al. (2017) found that patients see their ED as their identity and not as something that requires treatment and that readiness to change and improper delivery of therapy are factors that impact response to treatment.

Evidence‐based therapies such as CBT‐E, SSCM, MANTRA, CFT‐E (see Table 1) and schema therapy all have weight restoration as a primary focus, which patients with SEED may perceive as an unattainable goal. Instead, an overall consensus should focus on patient‐centred approaches, improving quality of life and giving patients autonomy over their care in terms of identifying goals which are meaningful to them. Reay et al. (2022) found that working on staying well enough so patients can stay out of hospital and engage in their chosen day‐to‐day activities, such as work and hobbies, was central for patients with EDs. Small achievable goals can be formulated to reduce ED behaviours to a safer level that reduces overall distress and minimises physical harm. This study found that psychoeducation remains an important part of treatment as it allows patients with capacity to make informed decisions about their care and equips individuals with the benefits of change to maximise their chance of a healthier and overall better quality of life. Studies identified how valuable OTs are for people with EDs, as they have specific skills in assessing individuals' ability to actively engage in meaningful daily activities (Fichter and Quadflieg 2016; Mack et al. 2023).

This study identified the importance of assessing and managing co‐morbidities such as trauma and autism when working with patients with SEED. Participants highlighted the potential benefits of integrating ED treatments with those targeting co‐morbidities in terms of improving quality of life and trauma‐related symptoms. Studies (Mitchell et al. 2021; Trottier and Monson 2021; Claudat et al. 2022; Brewerton 2023; Perlman 2023) highlighted the importance of understanding the function an ED may be serving in a person's life, for example, as a means of coping with underlying trauma. Key findings from this study is the need to develop an understanding of the factors contributing to a patient's ED and to have the flexibility within available treatments to respond to the individual needs of the patient. Interventions should not be segregated as all mental health challenges are interlinked, and an individual should be treated holistically. Participants highlighted that adequate training should be provided to increase confidence when addressing these complex co‐morbidities, particularly those with neurodiversity. Studies on autism and EDs (Kinnaird et al. 2017, 2020; Westwood and Tchanturia 2017; Babb et al. 2021) also highlighted the need to adapt interventions to take into account a patient's social communication and sensory differences.

It was evident from the findings of this study that HCPs acknowledge the frequent discrepancies between service‐providers' and patient's treatment goals. Participants described how services currently do not cater appropriately for patients with SEED because expectations are often too high with an emphasis on recovery and weight restoration. This study recognised the clear need to allow patients with SEED to choose their own goals, treatment options and live their lives the way they want, but current practices do not support this. Studies (Bianchi et al. 2021; Reay et al. 2022; Webb et al. 2022) also highlighted the ethical considerations associated with providing interventions that are not recovery focused and acknowledged the need for more flexibility in treatment options for this cohort.

The continued lack of clarity in relation to defining criteria for SEED has been identified as a challenge in the literature (Kotilahti et al. 2020). This study found that services are slow to recognise and respond to the needs of this cohort in a co‐ordinated way. They also emphasised the need for HCPs to receive regular supervision, receive specialised training to equip staff to effectively deliver evidence‐based interventions in a person‐centred way, and team‐building activities to support each other. Supportive and collaborative team working has been established as essential for providing care to SEED patients (Couturier et al. 2018).

### 5.1 Strengths and Limitations

This study builds upon limited empirical research that explored how HCPs are optimising PSIs for patients with SEED. A strength of this study was the recruitment of varied HCPs from nine different mental health services across Ireland. Drawing on these multiple perspectives provided enhanced understanding by locating the areas where HCPs' perspectives aligned and where they differed. However, the majority of the participants were female (*n* = 15:1), and thus, data may not adequately reflect a male perspective on these issues. All the participants had experience of working with patients with SEED and, therefore, were an adequate selection given the methods and scope of the study. To the best of our knowledge, this is the first study to consider HCPs' perceptions of using PSI with patients who have SEED in Ireland, which represents progress in developing knowledge in this area. Further research is needed on the effectiveness of PSI, in particular when treating co‐morbidities for patients with SEED and how to tailor these interventions to best meet their needs.

## 6 Conclusion

This study provides insight into HCPs' perspectives of delivering PSIs to patients with SEED. The findings expand upon previous research exploring the delivery of evidence‐based interventions to this client group and the challenges associated with managing the complex needs of these patients. Despite NICE guidelines (2017) recommending access to psychological interventions for people with SEED, there is a growing body of research which has demonstrated that individuals with EDs often do not receive an evidence‐based treatment. It has been hypothesised that there is a discrepancy between evidence‐based treatments and actual treatment delivery which may be linked in part to service‐based factors such as lack of resources, training, therapist lack of knowledge and negative attitudes. This study highlighted the clinical implications of the dearth of guidelines on evidence‐based clinical guidelines on the treatment of patients with SEED in Ireland. The findings emphasise the importance of having access to a range of interventions which can be tailored to meet the client's needs and can integrate treatment of any co‐morbidities if required. The involvement of the patient in terms of collaborative decision‐making and targeting interventions to their own goals was also proposed. In summary, the findings support the need for adequate resourcing, innovation and research into psychosocial intervention and support for HCPs in providing these specialised services.

## 7 Relevance for Clinical Practice

Recommendations for changes in clinical practice include that adequate training is provided to equip HCPs to recognise ED behaviours at an early stage. It was emphasised that all HCPs specialising in working with patients with ED are trained in specific psychosocial interventions for ED, including the need to be flexible and collaborative when formulating treatment goals for patients with SEED. HCPs also highlighted the need for training in assessing and working with trauma and autism to allow them to provide an integrated multi‐modal approach when treating co‐morbidities as opposed to treating them in isolation.

## Author Contributions

K.M. conceptualisation, formal analysis, investigation, data curation, writing – original draft preparation, writing – reviewing and editing and visualisation. A.‐M.B. conceptualisation, formal analysis, validation and writing – reviewing and editing. A.G. conceptualisation, formal analysis, investigation, validation, supervision and writing – reviewing and editing.

## Ethics Statement

Ethical approval was obtained from the Nursing Research Ethics Committee: Research Ethics committee CH09—REC no: 11/2023, The Rotunda Hospital Research Ethics Committee—REC no: 2024‐007, Sligo Research Ethics Committee—REC no: 989.

## Consent

The authors have nothing to report.

## Conflicts of Interest

The authors declare no conflicts of interest.

## Supporting information


File S1.

File S2.

## Data Availability

The data that support the findings of this study are available from the corresponding author upon reasonable request.
