# Supplementary material for: Optimising Psychosocial Interventions for People With Severe and Enduring Eating Disorders (SEED) Perspectives of Healthcare Professionals in Ireland: A Qualitative Study
Source: Int J Ment Health Nurs. 2025 Apr 7;34(2):e70037. doi: 10.1111/inm.70037 (PMC11976135; doi:10.1111/inm.70037)
Supplement: Supplementary file 1 — File S1. [file INM-34-0-s002.docx]

**Supplementary File 1: COREQ CHECKLIST**

| **COREQ CHECKLIST** | **Reported in Manuscript** |
| --- | --- |
| **Domain 1: Research team and reflexivity** |  |
| *Personal Characteristics* |  |
| 1. Interviewer/facilitator: Which author/s conducted the interview or focus group? | Yes. P5-6 |
| 2. Credentials: What were the researcher’s credentials? | Yes. This is in the Title Page 4 |
| 3. Occupation: What was their occupation at the time of the study? Registered Mental Health Nurse | Yes. This is in the Title Page 4 |
| 4. Gender: Was the researcher male or female? | Yes. This is in the Title Page 4 |
| 5. Experience and training: What experience or training did the researcher have? | Yes. P4 |
| *Relationship with participants* |  |
| 6. Relationship established: Was a relationship established prior to study commencement?  Yes, not a formal relationship, some participants may have known the names of, and met some of the research team over the course of their careers. | Yes, not a formal relationship, met some of the participants over the course of their careers and some were colleagues. |
| 7. Participant knowledge of the interviewer: What did the participants know about the researcher? | Job title and name of course. |
| 8. Interviewer characteristics: What characteristics were reported about the interviewer/facilitator? | Yes. P8-10 |
| **Domain 2: Study design** |  |
| *Theoretical framework* |  |
| 9. Methodological orientation and Theory: What methodological orientation was stated to underpin the study? | Yes. P5 |
| *Participant selection* |  |
| 10. Sampling: How were participants selected? | Yes. P5-7 |
| 11. Method of approach: How were participants approached? | Yes. P5-7 |
| 12. Sample size: How many participants were in the study? | Yes. P6 |
| 13. Non-participation: How many people refused to participate or dropped out? Reasons? | No. This information was not collected. |
| *Setting* |  |
| 14. Setting of data collection: Where was the data collected? | Yes. P5-7 |
| 15. Presence of non-participants: Was anyone else present besides the participants and researchers? | No, no one else was present |
| 16. Description of sample: What are the important characteristics of the sample? | Yes. P8-10 |
| *Data collection* |  |
| 17. Interview guide: Were questions, prompts, guides provided by the authors? Was it pilot tested? | Yes. P6-7 and Supplementary material file 2 |
| 18. Repeat interviews: Were repeat interviews carried out? If yes, how many? | No |
| 19. Audio/visual recording: Did the research use audio or visual recording to collect the data? | Yes, P6 |
| 20. Field notes: Were field notes made during and/or after the interview or focus group? | Yes. P6-7 |
| 21. Duration: What was the duration of the interviews or focus group? | P8 |
| 22. Data saturation: Was data saturation discussed? | No |
| 23. Transcripts returned: Were transcripts returned to participants for comment and/or correction? | No, but participants were given option to request. |
| **Domain 3: Analysis and findings** |  |
| *Data analysis* |  |
| 24. Number of data coders: How many data coders coded the data? | Yes. P7 |
| 25. Description of the coding tree: Did authors provide a description of the coding tree? | Yes. P7 |
| 26. Derivation of themes: Were themes identified in advance or derived from the data? | Derived from the data. P7 |
| 27. Software: What software, if applicable, was used to manage the data? | Yes. NVivo 14, P12 |
| 28. Participant checking: Did participants provide feedback on the findings? | No |
| *Reporting* |  |
| 29. Quotations presented: Were participant quotations presented to illustrate the themes / findings? Was each quotation identified? e.g. participant number | Yes. P11-18, and in Figure 1 |
| 30. Data and findings consistent: Was there consistency between the data presented and the findings? | Yes. P11-18 |
| 31. Clarity of major themes: Were major themes clearly presented in the findings? | Yes. P11-18 |
| 32. Clarity of minor themes: Is there a description of diverse cases or discussion of minor themes? | Yes within the relevant themes P11-22 |
