# Supplementary material for: Optimising Psychosocial Interventions for People With Severe and Enduring Eating Disorders (SEED) Perspectives of Healthcare Professionals in Ireland: A Qualitative Study
Source: Int J Ment Health Nurs. 2025 Apr 7;34(2):e70037. doi: 10.1111/inm.70037 (PMC11976135; doi:10.1111/inm.70037)
Supplement: Supplementary file 2 — File S2. [file INM-34-0-s001.docx]

**Supplementary File 2: Participant Interview Topic Guide**

**Experience working with SEED:**

Q1: Could you tell me about your experience of care with patients with SEED? – what are the characteristics of a person with SEED in your opinion?

Q2: What has been your experience of building therapeutic relationships with patients who have SEED? (Barriers and facilitators to the relationship?)

**Experience delivering interventions/evidence-based therapies:**

Q3: Can you tell me about your experience of providing psychosocial interventions for people with SEED?

Q4: Which psychosocial interventions do you currently deliver, or have you delivered for people with SEED?

Q5: In your opinion, what works well with existing psychosocial interventions that you deliver/have delivered for people with SEED? (e.g. i.t.o. effectiveness, recovery rate etc - Barriers and Facilitators

Q6 In your opinion, what works less well with existing psychosocial interventions that you deliver/have delivered for people with SEED?

Q7: What do you think helps to motivate HCP’s to use/implement psychosocial interventions with people with SEED? What are the challenges for staff?

Q8: In your opinion, how do we as HCP’s manage the physical health concerns associated with having SEED whilst providing a PSI? (talk about complexity in care?)

Q9: In your opinion, do the physical health concerns associated with having SEED affect the psychosocial intervention HCPs choose? If so, how?

Q10: What suggestions do you have about how to improve the psychosocial interventions for people with SEED? – how can we improve the interventions we are offering?

Q11: Are there any other aspects of your experience or thoughts regarding psychosocial interventions for people with SEED that you would like to tell us about?

**Working with co-morbidities:**

Q12: In your service/in your experience what assessment and treatment options do you use/provide for comorbidity in people with SEED? (i.e. trauma, PTSD, EUPD, autism, anxiety, depression)

Q13: What self-report measures do you use to learn more about the additional difficulties people with SEED are experiencing (e.g. BDI, BAI, GAD-7, PHQ-9, PCL-5, Revised Impact of Events Scale (IES-R), CRIES) and do you think the use of these measures should be prioritised to gain more understanding of patient challenges?

Q14: What else can healthcare professionals do to further support patients with SEED who have comorbidity? (such as trauma, PTSD, autism, EUPD, anxiety, OCD, depression)

Q15: What support, competency skills or training requirements do you consider would help you as HCP to help patients with comorbidities such as trauma, PTSD, autism, self-harm, OCD etc.

***Is there anything else you would like to add and say etc?***

***Thank everyone for participating.***
